# Duration and Predictive Factors of Plastic Biliary Stent Patency: Results of a Large Prospective Database Analysis

**DOI:** 10.3390/jcm14248788

**Published:** 2025-12-11

**Authors:** Egle Dieninyte, Eugenijus Jasiunas, Aistis Lemezis, Emilija Kezeviciute, Juozas Stanaitis, Tomas Poskus

**Affiliations:** 1 Institute of Clinical Medicine, Faculty of Medicine, Vilnius University, 01513 Vilnius, Lithuania; 2 Vilnius University Hospital Santaros Klinikos, 08406 Vilnius, Lithuania

**Keywords:** endoscopic retrograde cholangiopancreatography, biliary stent patency, stent occlusion, cholangitis

## Abstract

**Background/Objectives**: Endoscopic retrograde cholangiopancreatography (ERCP) with biliary stent placement is a mainstay of current management for biliary obstruction, with stent occlusion being the most common and severe complication. The mechanism of stent occlusion is well known; however, factors affecting individual stent patency are still controversial. The objective of this study was to determine the duration and factors affecting plastic biliary stent patency. **Methods**: We conducted a retrospective analysis of the consecutive procedures of endoscopic retrograde cholangiopancreatography (ERCP) and biliary stent placement in a single tertiary center during the period of 2010–2019. The primary outcome of the study was the time of stent patency. Secondary outcomes were the development of cholangitis upon re-stenting and whether subsequent re-stenting was emergent. Re-stenting was considered *emergent* if it happened before the planned elective re-stenting date, irrespective of indication (development of cholangitis, rising jaundice, suspected dislodgement, etc.). **Results**: Between 2010 and 2019, a total of 5462 ERCP procedures were performed, with 2659 resulting in plastic biliary stent placement. On average, the plastic biliary stent was patent for 63 (25, 96) days with significant differences between the indications for ERCP groups. The strongest risk factors for the development of cholangitis upon re-stenting was cholangitis during index ERCP (HR = 1.83; 95% CI: 1.48–2.27; *p* < 0.001), intrabiliary malignancy being the indication for stenting (HR = 1.34; 95% CI: 1.12–1.60; *p* < 0.001) and increasing number of stents being placed (HR = 1.73; 95% CI: 1.27–2.36; *p* < 0.001). **Conclusions**: Patients with an underlying malignancy, history of cholangitis, and multiple biliary stents are at an increased risk for stent occlusion and cholangitis, warranting a tailored stent exchange interval to prevent complications.

## 1. Introduction

Endoscopic retrograde cholangiopancreatography (ERCP) with biliary stent placement is a mainstay of current management of patients with both malignant and benign biliary obstruction. While plastic stents are favored for their ease of placement, removal, and cost effectiveness, their durability remains limited—typically maintaining patency for only 2–4 months, with reported medians ranging from ~55 days in malignant disease to ~110 days in benign conditions [1,2].

It has long been known that obstruction of plastic stents most commonly results from bacterial biofilm formation, proteinaceous sludge accumulation, and tumor or tissue ingrowth. These processes are influenced by both stent-related and patient-specific factors [3,4,5]. Studies have shown that malignant obstruction, particularly perihilar, significantly shortens stent patency [1]. Furthermore, clinical variables such as elevated bilirubin levels, multiple stents, and hypoalbuminemia have been shown to increase the risk of subsequent cholangitis [6,7,8]. In one large retrospective cohort, patients with hilar malignancy were at a three-fold increased risk of early stent dysfunction compared to those with distal strictures [9].

The mechanism of the biofilm formation on a plastic stent resulting in occlusion is well known; however, there remains a lack of comprehensive models that integrate procedure-related, stent-related, and patient variables to predict stent occlusion and complications. The aim of this study was to investigate the duration of plastic biliary stent patency and risk factors for stent occlusion.

## 2. Materials and Methods

A retrospective analysis of a prospectively collected database of ERCP procedures carried out between 2010 and 2019 in a single tertiary care hospital in Vilnius, Lithuania, was performed. Permission by the regional bioethics committee for the study was obtained (permission number 2023/10-1539-1006). The study was conducted in accordance with the principles of the Declaration of Helsinki.

All procedures that resulted in a plastic biliary stent placement were included in the analysis of plastic biliary stent patency. Patients who did not require stent placement, such as patients after dilation of benign strictures, successful choledocholithiasis, or patients who were stented with metal stents, were excluded from the analysis.

The primary outcome of the study was the duration of the stent patency, considered as the time elapsed between index stent placement and the subsequent re-stenting. Secondary outcomes were the development of cholangitis upon repeated stenting and whether subsequent re-stenting was emergent. Re-stenting was considered *emergent* if it happened before the planned elective re-stenting date, irrespective of indication (development of cholangitis, rising jaundice, suspected dislodgement, etc.).

To evaluate the impact of different etiologies of biliary obstruction on stent patency, patients were divided into four groups: patients with strictures due to extrabiliary malignancy (pancreatic cancer, metastatic compression, etc.), intrabiliary malignancy (cholangiocarcinoma), benign biliary strictures (chronic pancreatitis, post-cholecystectomy strictures, etc.) and biliary strictures due to parasitic infestation (echinococcal infection). All patients received routine antibiotic prophylaxis according to the hospital guidelines on the day of ERCP. Administration of additional antibiotics upon hospitalization was considered as use of antibiotics.

Differences between groups were evaluated using the Kruskal–Wallis test for continuous variables, Pearson’s Chi-squared test, or Fisher’s exact test for categorical variables. Effect size was calculated using Cramer’s V adjusted (for nominal variables) or Rank epsilon squared (for interval variables) statistics. To estimate stent patency time (survival function), Kaplan–Meier curves were plotted that were compared using the log-rank test. To assess risk factors for repeated stenting being performed as an emergency (non-elective) procedure and development of cholangitis multivariate Cox proportional hazards regression model was used. A two-sided *p*-value of less than 0.05 indicated statistical significance. Statistical analyses were conducted using R software, version 4.5.0 (R Project for Statistical Computing).

## 3. Results

### 3.1. Patient Population and Characteristics

Between 2010 and 2019, a total of 5462 ERCP procedures were performed. Out of the total, 2659 ERCP procedures resulted in plastic biliary stent placement and were included in this study. In total, 1452 (56.4%) of the patients were male, and the average patient age was 64 ±15 years. A total of 2659 patients were followed up and included in the present study. The majority of biliary stent placements were indicated for extrabiliary malignancies (N = 1041, 39.2%). All of the baseline characteristics between the biliary stricture’s etiology groups differed with statistical significance (*p* < 0.001). Patients with both extrabiliary and intrabiliary malignancies were older (67 ± 12 years and 68 ± 12 years, respectively, compared to patients with benign strictures, 60 ± 17 years, and parasitic infestation, 52 ± 16 years). The same trend was detected regarding the presence of cholangitis, most often present in the extrabiliary malignancy group (N = 400, 38%), followed by the intrabiliary malignancy group (N = 318, 31%), benign stricture (N = 243, 23%) and parasitic infestation group (N = 81, 7.8%). The highest number of stents was placed in intrabiliary malignancy and parasitic infestation groups (1.55 ± 0.57 and 1.56 ± 0.55, respectively). Baseline characteristics of the patients are shown in Table 1.

Patients with malignant indications for biliary stenting had to undergo emergent re-stenting more often (N = 243, 39%; N = 294, 33%; N = 175, 20% and N = 73, 8% for extrabiliary and intrabiliary malignancy, benign stricture and parasitic infestation groups, respectively). A similar tendency regarding diagnosis of cholangitis during the subsequent re-stenting was observed, in groups of malignant indication for ERCP incidence being higher (N = 287, 37%; N = 293, 38%; N = 125, 16% and N = 69, 9% for extrabiliary and intrabiliary malignancy, benign stricture, and parasitic infestation groups, respectively).

### 3.2. Primary Outcome

**Figure 1:**
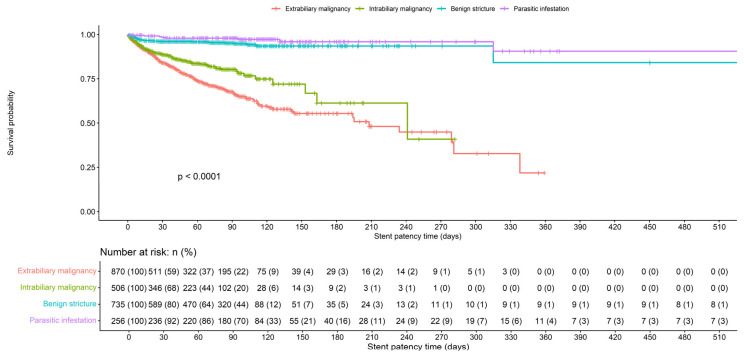
Plastic biliary stent patency time.

On average, the plastic biliary stent was patent for 63 (25, 96) days. There were significant differences in stent patency duration between different indications for ERCP groups (Figure 1). The shortest stent patency time was observed in the extrabiliary malignancy group, 41 (17, 84) days, while the stent was patent the longest in cases of parasitic infestation, 98 (84, 136) days. The log-rank test confirmed that the difference in stent patency time between etiological groups is statistically significant (*p* < 0.0001).

### 3.3. Secondary Outcomes

Overall, cholangitis during the subsequent re-stenting was diagnosed in 774 cases (33%).

The strongest risk factors for the development of cholangitis upon the subsequent re-stenting was found to be cholangitis during the index ERCP (HR = 1.83; 95% CI: 1.48–2.27; *p* < 0.001), intrabiliary malignancy being the indication for stenting (HR = 1.34; 95% CI: 1.12–1.60; *p* < 0.001) and increasing number of stents being placed (HR = 1.73; 95% CI: 1.27–2.36; *p* < 0.001). The risk for the diagnosis of cholangitis during the subsequent re-stenting was also slightly increased in male patients (HR = 1.2, 95% CI: 1.04–1.40; *p* = 0.0150).

**Figure 2:**
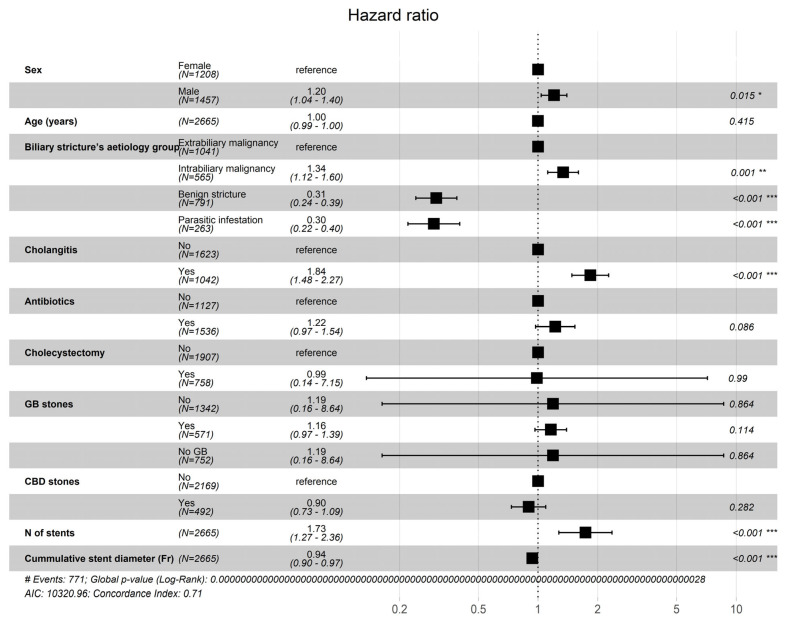
Risk factors for presenting with cholangitis during the subsequent re-stenting. Significance codes: *—0.01, **—0.001, ***—0.

Two factors that were related to the reduced risk of subsequent diagnosis of cholangitis were identified: indication for biliary stenting being benign stricture (HR = 0.31; 95% CI: 0.24–0.39; *p* < 0.001) and parasitic infestation (HR = 0.30; 95% CI: 0.22–0.40; *p* < 0.001) (Figure 2).

In total, 885 (33%) subsequent re-stentings were performed as an emergency (non-elective). The strongest risk factors for successive for ERCP being non-elective on multivariate Cox regression analysis were increasing number of biliary stents placed (HR = 1.49; 95% CI: 1.11–1.99; *p* < 0.001), prescription of antibiotics and presence of cholangitis during the index ERCP (HR = 1.45; 95% CI: 1.20–1.77; *p* < 0.001 and HR = 1.29; 95% CI: 1.07–1.55; *p* < 0.001). The risk was also increased in patients with intrabiliary malignancy (HR = 1.22; 95% CI: 1.03–1.45; *p* = 0.02).

**Figure 3:**
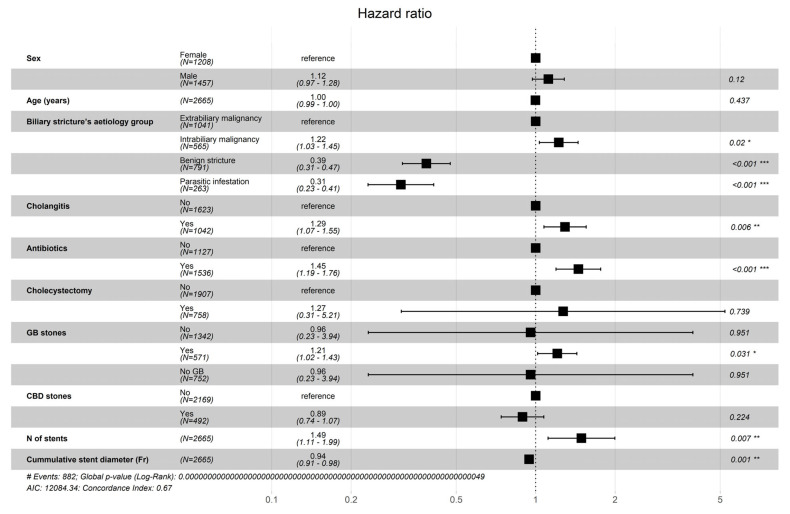
Risk factors for the subsequent re-stenting are emergent. Significance codes: *—0.01, **—0.001, ***—0.

Two factors were significantly associated with a reduced risk of successive cholangitis on multivariate Cox regression analysis: indication for biliary stenting being benign stricture (HR = 0.39; 95% CI: 0.31–0.48; *p* < 0.001) and parasitic infestation (HR = 0.31; 95% CI: 0.23–0.41; *p* < 0.001). Increasing cumulative biliary stent diameter slightly decreased the risk of cholangitis development (HR = 0.94; 95% CI: 0.91–0.98; *p* < 0.001 (Figure 3).

## 4. Discussion

This study into plastic biliary stent patency and the risk factors affecting it is the biggest analysis of this question to date. It shows an average duration of stent patency of 63 days, with a significant impact of the indication for biliary stent placement on the stent patency. In addition, it identifies risk factors for complications, such as diagnosis of cholangitis at the index ERCP, increasing number of plastic stents placed.

Our results show that patients with a malignancy as an indication for biliary stent placement have a shorter plastic stent patency than those with a benign indication. This is concordant with previously published data reporting reduced stent patency time in patients with malignancy (106 vs. 55 days for malignant and benign underlying disease, respectively) [1]. This can be explained by the progressive course of disease in cases of malignancy and increased pressure in the biliary tract.

Although it is known that the time of a plastic biliary stent patency is not sufficient to complete chemotherapy in a neoadjuvant setting [10], the effect of chemotherapy seems to prolong stent patency of metal stents in a palliative setting [11], likely resulting from tumor shrinkage and decreased biliary sludge.

Our data showed slightly longer stent patency in the intrabiliary malignancy group compared to the extrabiliary malignancy group. This is an unexpected finding since a significant number of patients with intrabiliary malignancies had perihilar strictures, usually requiring placement of multiple smaller-diameter plastic stents. It is also in contrast with currently published data. Ostrowski et al. [1] investigated differences in stent patency within malignancies, showing decreased stent patency for proximal stricture location (40 vs. 76 days for perihilar and distal strictures, respectively). The longer stent patency time in the intrabiliary malignancy group compared to the extrabiliary malignancy group is also opposed by the finding that diagnosis of intrabiliary malignancy is a risk factor for the development of cholangitis. This discrepancy could have arisen due to a couple of reasons. Per our center’s protocol, all patients undergoing ERCP and biliary stenting with plastic stents are scheduled for an elective re-stenting in 3 months. Any hospital admission and subsequent re-stenting, whether due to rising jaundice that could impede chemotherapy course or cholangitis, was regarded as an emergent case in this study. In the case of perihilar cholangiocarcinoma, dislodgement of longer stents is more common [12] and can result in localized biliary obstruction without cholangitis, with subsequent decision to emergently re-stent. Moreover, considering patients with perihilar cholangiocarcinoma and stent placement in the left and/or right hepatic ducts, obstruction of one of the stents might result in focal cholangitis without apparent systemic symptoms of cholangitis.

Not surprisingly, the longest stent patency was observed in the parasitic infestation group. Although rare, Echinococcal infection is endemic in certain parts of the world, and this study provides the most comprehensive analysis of outcomes of biliary stent patency in this parasitic infestation. The simplest explanation is that patients with parasitic infestations are on a constant antibiotic therapy that targets Gram-negative bacteria, thus inhibiting one of the major mechanisms in biofilm formation and subsequent stent occlusion—bacterial colonization [5]. A more hypothetical explanation could raise a question, whether the destructive secretions of Echinococcus species that enable aggressive tissue invasion might affect the biofilm formation in plastic biliary stents.

We have found that the risk of cholangitis development was higher in patients with intrabiliary malignancies, presenting with cholangitis upon index procedure, and increased with multiple stents being placed. These findings are similar to currently published data, showing that the diagnosis of malignancy, placement of multiple biliary stents, prior occlusion events and male gender are risk factors for the development of cholangitis after plastic stent placement [2,7]. In contrast to our findings, a dedicated study of 51 patients with hepatic alveolar echinococcosis undergoing ERCP reported a similar rate of cholangitis (9.1%), but identified placing a single plastic stent or a stent diameter of >8.5 Fr as risk factors for cholangitis [13].

In addition to the identified risk factors for the development of cholangitis, the influence of the quantity of plastic stents and their cumulative diameter was even higher, considering the risk for the subsequent re-stenting being non-elective. The risk of occlusion increases with multiple number of plastic stents being placed and decreases as the cumulative stent diameter increases. Placing multiple smaller-diameter plastic stents increases the surface area of the stent for biofilm formation and decreases lumen diameter, resulting in earlier occlusion. This principle is clearly observed in the superior patency of larger-diameter metal stents.

Recently published studies into cultural and metagenomic analysis of bile and biliary stents have led to plastic biliary stents identifying possible specific microbial drivers of stent occlusion and hold promise for tailored approaches and plastic biliary stent innovations to prolong their patency [14,15].

Although being robust in patient inclusion, our study has several limitations, mostly due to its retrospective nature. A tenth of our cohort patients, mostly with underlying malignancies, were lost to follow up resulting in a possible bias in the results. Another limitation is the crudeness of data collected in the database, not accounting for possible concomitant factors, possibly affecting stent patency, such as chemotherapy, laboratory workup, comorbidities, etc. This is important considering stent patency time in patients diagnosed with cholangiocarcinoma. Recent publications have shown that stent patency is significantly shorter in cases of perihilar cholangiocarcinoma compared to distal biliary cancer [16], but due to electronic database features, this subgroup analysis was not possible. Despite these shortcomings, a significant number of patients in each patient cohort seem to indicate robust differences between the groups and provide reliable data on the factors analyzed.

## 5. Conclusions

Patients with an underlying malignancy, history of cholangitis and multiple biliary plastic stents are at an increased risk for stent occlusion and cholangitis. A more vigilant and tailored stent exchange interval, based on the underlying disease, previous occlusion events and procedural aspects is warranted to prevent complications.

## Figures and Tables

**Table 1:** Baseline characteristics of the patients.

Characteristic	Overall * N = 2659	Biliary Stricture’s Etiology Group				*p*-Value **	Effect Size (ES) Adjusted (95% CI) ***
		Extrabiliary Malignancy * N = 1041 (39%)	Intrabiliary Malignancy * N = 565 (21%)	Benign Stricture * N = 790 (30%)	Parasitic Infestation * N = 263 (10%)		
Sex (male)	1452 (55%)	456 (44%)	271 (48%)	523 66%	202 77%	<0.001	0.24 (0.20, 1.0)
Age (years) ^^^	64 (15)	67 (12)	68 (12)	60 (17)	52 (16)	<0.001	0.10 (0.08, 1.0)
Cholangitis ^+^	1042 (39%)	400 (38%)	318 (31%)	243 (23%)	81 (7.8%)	<0.001	0.19 (0.16, 1.0)
Antibiotics °	1533 (58%)	577 (38%)	400 (26%)	443 (29%)	113 (7.4%)	<0.001	0.15 (0.12, 1.0)
Cholecystectomy ^Δ^	756 (28%)	196 (26%)	125 (17%)	405 (54%)	30 (4.0%)	<0.001	0.33 (0.30, 1.0)
Presence of gallbladder stones	570 (21%)	228 (40%)	145 (25%)	148 (26%)	49 (8.6%)	<0.001	0.24 (0.22, 1.0)
Presence of CBD stones	492 (19%)	88 (18%)	72 (15%)	243 (49%)	89 (18%)	<0.001	0.27 (0.24, 1.0)
N of stents ^^^ placed	1.34 (0.55)	1.13 (0.36)	1.55 (0.57)	1.37 (0.63)	1.56 (0.55)	<0.001	0.13 (0.11, 1.0)
Cumulative stent diameter (Fr) ^±^	12.0 (10.0, 17.0)	12.0 (10.0, 12.0)	14.0 (10.0, 17.0)	12.0 (10.0, 17.0)	14.0 (10.0, 17.0)	<0.001	0.04 (0.03, 1.0)
Stent patency time (days) ^±^	63 (25, 96)	41 (17, 84)	54 (22, 84)	83 (38, 100)	98 (84, 136)	<0.001	0.12 (0.10, 1.0)
Stent patency >30 days	1680 (63%)	509 (30%)	346 (21%)	589 (35%)	236 (14%)	<0.001	0.25 (0.22, 1.0)
Next re-stenting emergency	885 (33%)	343 (39%)	294 (33%)	175 (20%)	73 (8%)	<0.001	0.26 (0.22, 1.0)
Cholangitis during the next re-stenting	774 (29%)	287 (37%)	293 (38%)	125 (16%)	69 (9%)	<0.001	0.31 (0.28, 1.0)
**Survival**						<0.001	0.21 (0.18, 1.0)
Alive	2021 (76%)	650 (32%)	421 (21%)	702 (35%)	248 (12%)		
Dead	348 (13%)	220 (63%)	85 (24%)	35 (10%)	8 (2.3%)		
Unknown	289 (11%)	171 (59%)	59 (20%)	52 (18%)	7 (2.4%)		

* N (%), ** Kruskal–Wallis test; Pearson’s Chi-squared test, Fisher’s exact test, *** Cramer’s V adjusted, Rank epsilon squared. ^^^ Continuous variable of normal distribution defined by mean and standard deviation. ^±^ Continuous variable of non-normal distribution defined by median and interquartile range. ^+^ Cholangitis—diagnosed based on diagnostic criteria upon time of hospitalization. ° Antibiotics—administration of additional antibiotics upon hospitalization, other than routine antibiotic prophylaxis for ERCP procedure. ^Δ^ Cholecystectomy—performance of cholecystectomy in patient’s history prior to hospitalization.

## Data Availability

Anonymized raw data presented in this study are available on request from the corresponding author due to restrictions on the permission by the regional bioethics committee.
